# Cardio-Respiratory Reference Data in 4631 Healthy Men and Women 20-90 Years: The HUNT 3 Fitness Study

**DOI:** 10.1371/journal.pone.0113884

**Published:** 2014-11-26

**Authors:** Henrik Loe, Sigurd Steinshamn, Ulrik Wisløff

**Affiliations:** 1 K.G. Jebsen Center of Exercise in Medicine at Department of Circulation and Medical Imaging, Norwegian University of Science and Technology, Trondheim, Norway; 2 Valnesfjord Rehabilitation Center, Valnesfjord, Norway; 3 Department of Thoracic Medicine, St. Olavs Hospital, Trondheim University Hospital, Trondheim, Norway; VU University Medical Center, Netherlands

## Abstract

**Purpose:**

To provide a large reference material on key cardio-respiratory variables in a healthy population of Norwegian men and women aged 20–90 years.

**Methods:**

Sub maximal and peak levels of cardio-respiratory variables were measured using cardiopulmonary exercise testing during treadmill running.

**Results:**

The highest peak ventilation among men (141.9±24.5 L·min^−1^) and women (92.0±16.5 L·min^−1^) was observed in the youngest age group (20–29 years, sex differences p<0.001) with an average 7% reduction per decade. The highest tidal volumes were observed in the 30–39 and 40–49 year age groups among men (2.94±0.46 L) and women (2.06±0.32 L) (sex differences p<0.001), with a subsequent average 6% reduction per decade. Ventilatory threshold and respiratory compensation point were observed at approximately 77% and 87% of peak oxygen uptake (VO_2peak_) among men and women, respectively. The best ventilatory efficiency (EqVCO_2Than_) was observed in the youngest age group (20–29 years) in both men (26.2±2.8) and woman (27.5±2.7) (sex differences p<0.001) with an average 3% deterioration in ventilatory efficiency per decade.

**Conclusion:**

This is the largest European reference material of cardio-respiratory variables in healthy men and women aged 20–90 years, establishing normal values for, and associations between key cardio-respiratory parameters. This will be useful in clinical decision making when evaluating cardiopulmonary health in similar populations.

## Introduction

Cardiopulmonary exercise testing (CPET) is an underutilized clinical procedure [1], despite several published recommendations by international health organizations [2]–[5]. It is a good tool for evaluating mechanisms and limitations of exercise tolerance [6], and for the assessment of cardiopulmonary health [1]. Consequently, CPET is a valuable tool in clinical decision making [2]. There are numerous large studies establishing spirometry reference values [7]–[14]. However, studies containing a combination of principal cardio- respiratory variables, such as ventilation (V_E_), tidal volume (V_T_), breathing frequency (f_B_), oxygen uptake (VO_2_), expired carbon dioxide (VCO_2_) and ventilatory efficiency (EqVCO_2_) are limited. Previous research have included only a few of these key variables, are limited to selected age groups and fitness levels, or small populations e.g. [15]–[25]. The aim of this study was to establish a large reference material on submaximal and maximal cardiopulmonary variables in healthy men and women aged 20–90 years.

## Methods

### Participants

The HUNT 3 fitness study is the third wave of the Nord-Trøndelag Health Studies (ntnu.edu/hunt). Data were collected between October 2006 and June 2008. The entire population >20 years of age (n = 94194) were invited to participate, 54% (n = 50821) accepted. Eligible candidates had to be free from cardiovascular disease, respiratory symptoms, cancer, and the use of blood pressure medication. Based upon self-reported information, 30513 candidates presented as suitable for VO_2max_ testing. Out of these, 12609 candidates resided in the 3 municipals selected for VO_2max_ testing, and 5633 of them volunteered to participate. Subsequent the primary inclusion the medical interview excluded an additional 390 candidates not meeting medical inclusion criteria, leaving 5243 candidates. 4631candidates completed a VO_2peak_ test. These 3 locations were chosen due to geographical location to minimize travel distance for participants. We experienced technical difficulties with Cortex MetaMax ∏, and during service at Cortex data was lost, thus, total sample sizes on tidal volume (V_T_) and breathing frequency (f_B_) was n = 3667.

### Ethics statement

The study was approved by the Regional committee for medical research ethics (2012/1672/REK nord), the Norwegian Data Inspectorate and the National Directorate of Health, and is in compliance with the Helsinki declaration. Written informed consent was obtained from all participants.

### Cardio Pulmonary Exercise Test (CPET)

An individualized graded protocol [26] was used for measuring cardio-respiratory variables (Cortex MetaMax ∏, Cortex, Leipzig, Germany). Before starting the testing procedure several MetaMax II apparatus were tested against Douglas-bag and iron lung (Cortex, Leipzig, Germany) and those finally used found reliable and valid [27]. Speed and angle of the test treadmills were calibrated prior to testing. The MetaMax II was calibrated prior to the first test each day using a standard two-point gas calibration procedure. The calibration included measurements of ambient air and a gas mix of known content (15.03% O_2_ and 4.98% CO_2_ in N_2_), a calibration of the Triple-V volume transducer with a calibrated 3 L syringe (Calibration syringe D, SensorMedics, CareFusion, San Diego, CA, USA), and barometric pressure control. Volume calibration was performed every third test and the two-point gas calibration every fifth. Before each test the ambient room air was checked. Heart rate was measured by radio telemetry (Polar S610i, Polar Electro Oy, Kempele, Finland). Body mass was measured using the weighing scale Model DS-102 (Arctic Heating AS, Nøtterøy, Norway). Participants had a treadmill familiarization phase of 8–10 minutes during warm-up. They were instructed to avoid grabbing the handrails if not absolutely necessary. The individualized warm-up workload determined the initial speed/angle on the subsequent treadmill test. Candidates used a face mask (Hans Rudolph, Germany) of appropriate size linked to the MetaMax ∏. When participant maintained a stable oxygen uptake >30seconds, velocity (0.5–1.0 kmh-1) or inclination (1–2%) was increased. Increased workload was if possible obtained with increased speed and keeping a fixed slope angle of the treadmill. If a participant was unable to increase speed, the angle was increased. Tests were ended when candidates reached volitional exhaustion (e.g. shortness of breath and leg fatigue). VO_2max_ was considered achieved if subjects reached a VO_2_ plateau that remained stable even with increased work load [28], i.e. VO_2_ did not increase more than 2 mL·kg^−1^·min^−1^ despite increased work load, and R≥1.05. Since 12.6% of the subjects failed to reach VO_2max_ we used the expression VO_2peak_. Measurements were done at 3 different workloads, 2 submaximal and peak. Level 1: The individual initial workload was determined during warm-up, and stable VO_2_ and heart rate were reached after 3 minutes. Level 2: Treadmill gradient was increased by 2% or speed increased 1 km•h^−1^, with steady state obtained after 2–3 minutes. Peak workload is described above.

### Ventilatory anaerobic threshold (V_Than_) and respiratory compensation point (RCP)

The transition point from aerobic to anaerobic metabolism denotes V_Than_ [29]. With increasing workload aerobic metabolism alone cannot provide the energy required, and energy production must increasingly depend upon anaerobic metabolism [30]. RCP marks the respiratory compensation for metabolic acidosis, beneath which V_E_ is strongly linked to VCO_2_, whereas above V_E_ increases faster relative to VCO_2_ [31], hence, onset of hyperventilation [32]. Both V_THan_ and RCP were established by the V-slope method [31].

### Ventilatory efficiency

We calculated the ventilatory equivalent EqVO_2_ (V_E_·VO_2_^−1^) and EqVCO_2_ (V_E_·VCO_2_^−1^) at VO_2peak_ and V_Than_. The ventilatory equivalents describe the fraction of minute ventilation (V_E_) to oxygen uptake (VO_2_), or to expired carbon dioxide (VCO_2_).

### Statistical analysis

Parametric analysis was used and QQ-plots supported the assumption of normally distributed data. Data are presented as arithmetic mean ± standard deviation. Analysis of variance (Anova) was used to determine differences between age groups. If a significant F-ratio was achieved, post hoc evaluations were completed using Bonferroni tests. An Independent-Samples T-test was used for establishing level of significance between sexes. Linear regression and curve linear regression, with 95% confidence interval, were used to test associations between cardio-respiratory parameters. Multiple linear regressions were used to generate prediction models. All statistical tests were two-sided. SPSS 20.0 (Statistical package for Social Sciences, Chicago; Illinois, USA), and GraphPad Prism 4.01 (GraphPad Software, San Diego, California, USA) were used to analyze data. Correlations were done using data from Level 1, Level 2 and peak as described above. A p-value of <0.05 was considered statistically significant.

## Results

Descriptive characteristics for men and women are presented in Table 1.

**Table 1:** Descriptive data for men and women in the HUNT 3 fitness study.

	Men	Women
	(n = 2261)	(n = 2370)
Age (yr.)	48.9±13.5	48.0±13.7
Anthropometrical data		
Height (cm)	179.2±6.5	165.9±5.9
Weight (kg)	85.6±11.5	69.8±11.2
BMI (kg•m^−2^)	26.6±3.2	25.4±3.9
Smoking status		
Never smoked/Quit smoking (%)	50.9/27.5	49.1/27.4
Daily smoker/Occasional smoker (%)	10.1/11.5	13.1/10.4

Data are presented as arithmetic mean ± SD. BMI: bod mass index.

### Peak ventilation (V_Epeak_) and tidal volume (V_Tpeak_)

Women had a 34% (p<0.001) lower V_Epeak_ than men. V_Epeak_ was similar and highest in the two youngest age groups (20–29 and 30–39 years) in both men and women. In women V_Epeak_ became 8% (p<0.01) lower per decade from age 30–39 up to 60–69 years. Compared to men aged 20–39 years, men aged 40–49 years had 3.5% (p<0.05) lower V_Epeak_, whereupon it decreased by 9% (p<0.001) per decade up to those aged 60–69 years. For both sexes we observed an average 16% (p<0.001) lower V_Epeak_ between the 2 most senior age groups (60–69 vs. +70 years) (Table 2).

**Table 2:** Physiological cardio-respiratory variables in the HUNT 3 Fitness study stratified by intensity levels, sex and age groups.

	Level 1 (<V_Than_)		Level 2 (<RCP)		Peak	
	Men	Women	Men	Women	Men	Women
**All**	(n = 999)	(n = 1020)	(n = 921)	(n = 99)	(n = 1758)	(n = 1754)
Workload (Watts)	99.2±24.2	68.6±19.0	115.1±24.8	81.7±19.9	177.2±37.0	119.0±25.0
f_c_ (beats·min^−1^)	141±19	149±19	153±19	160±18	180±16	179±15
%f_cpeak_	78.8±8.0	83.3±7.5	85.6±7.3	89.6±6.5		
%VO_2peak_	67.5±11.2	72.4±10.6	74.9±10.7	79.8±9.9		
f_B_ (1·min^−1^)	26±5.2	28±5	30±6	31±6	44±8	42±8
V_E_ (L·min^−1^)	60.8±14.6	45.1±10.7	73.7±19.6	54.6±14.2	123.7±25.7	81.8±17.6
V_T_ (V_E_·f_B_^−1^)	2.32±0.46	1.61±0.32	2.51±0.47	1.76±0.33	2.84±0.49	1.94±0.33
VCO_2_ (L·min^−1^)	2.14±0.50	1.54±0.36	2.54±0.58	1.82±0.41	4.22±0.92	2.76±0.62
VO_2_ (L·min^−1^)	2.46±0.56	1.77±0.38	2.78±0.61	1.98±0.42	3.74±0.76	2.47±0.51
R (CO_2_·VO_2_^−1^)	0.89±0.06	0.88±0.06	0.94±0.08	0.95±0.08	1.12±0.07	1.11±0.08
BMI	26.6±3.2	25.4±3.9				
**20–29 years**						
	(n = 80)	(n = 77)	(n = 66)	(n = 66)	(n = 152)	(n = 160)
Workload (Watts)	109.5±24.0	78.1±15.0	125.5±22.9	90.9±13.3	197.2±40.2	126.3±24.4
f_c_ (beats·min^−1^)	158±20	164±20	172±14	176±16	196±11	194±9
%f_cpeak_	80.8±8.8	85.0±8.3	87.8±5.4	0.7±6.5		
%VO_2peak_	64.5±12.5	68.3±10.9	73.1±10.7	6.3±9.6		
f_B_ (1·min^−1^)	29±6	30±5	32±6	34±7	50±9	47±7
V_E_ (L·min^−1^)	61.3±15.7	45.4±10.8	75.1±19.3	57.9±15.9	141.9±24.5	92.0±16.5
V_T_ (V_E_·f_B_^−1^)	2.14±0.41	1.53±0.31	2.39±0.46	1.73±0.31	2.84±0.52	1.91±0.31
VCO_2_ (L·min^−1^)	2.31±0.60	1.62±0.39	2.87±0.66	1.98±0.40	4.94±0.87	3.17±0.57
VO_2_ (L·min^−1^)	2.72±0.67	1.92±0.42	3.16±0.72	2.20±0.41	4.30±0.73	2.77±0.47
R (CO_2_·VO_2_^−1^)	0.90±0.06	0.89±0.06	0.96±0.05	0.97±0.08	1.15±0.06	1.14±0.06
BMI	24.2±3.1	23.9±3.9				
**30–39 years**						
	(n = 137)	(n = 191)	(n = 129)	(n = 167)	(n = 280)	(n = 326)
Workload (Watts)	109.3±21.9	76.7±16.9	123.1±22.0	90.9±19.3	196.8±32.6	127.4±23.9
f_c_ (beats·min^−1^)	149±17	157±17	163±16	169±16	190±9	188±11
%f_cpeak_	78.9±7.4	83.3±6.9	86.0±6.4	90.3±5.8		
%VO_2peak_	62.1±10.1	69.1±10.1	71.0±10.1	78.1±9.9		
f_B_ (1·min^−1^)	26±5	29±5	30±7	32±6	47±8	45±7
V_E_ (L·min^−1^)	61.7±15.3	47.3±12.2	76.4±20.1	58.6±15.3	136.8±21.5	91.5±15.5
V_T_ (V_E_·f_B_^−1^)	2.36±0.45	1.66±0.35	2.56±0.45	1.82±0.35	2.94±0.46	2.06±0.32
VCO_2_ (L·min^−1^)	2.31±0.53	1.66±0.40	2.75±0.54	1.98±0.42	4.82±0.75	3.13±0.57
VO_2_ (L·min^−1^)	2.63±0.52	1.91±0.40	2.98±0.52	2.16±0.44	4.20±0.65	2.74±0.50
R (CO_2_·VO_2_^−1^)	0.89±0.06	0.88±0.06	0.95±0.08	0.96±0.08	1.14±0.06	1.14±0.06
BMI	27.3±3.5	25.2±4.0				
**40–49 years**						
	(n = 267)	(n = 258)	(n = 253)	(n = 240)	(n = 469)	(n = 441)
Workload (Watts)	106.2±21.5	74.2±15.1	123.0±21.8	87.7±16.0	187.5±32.9	124.2±22.5
f_c_ (beats·min^−1^)	144±18	151±16	157±18	163±15	183±12	182±11
%f_cpeak_	78.6± 7.7	83.1±7.1	85.7±7.3	90.0±6.0		
%VO_2peak_	65.6±10.5	70.1±10.3	74.0±10.2	78.8±9.8		
f_B_ (1·min^−1^)	27±5	29±5	30±7	32±6	44±8	44±8
V_E_ (L·min^−1^)	63.1±14.8	47.3±10.1	78.1±21.0	58.3±13.7	132.0±22.1	87.7±14.8
V_T_ (V_E_·f_B_^−1^)	2.41±0.46	1.67±0.30	2.63±0.47	1.83±0.31	2.97±0.49	2.02±0.31
VCO_2_ (L·min^−1^)	2.26±0.45	1.62±0.34	2.70±0.51	1.95±0.41	4.54±0.73	2.96±0.53
VO_2_ (L·min^−1^)	2.62±0.56	1.85±0.35	2.97±0.59	2.10±0.39	4.00±0.62	2.62±0.44
R (CO_2_·VO_2_^−1^)	0.89±0.05	0.88±0.06	0.95±0.07	0.95±0.08	1.13±0.06	1.13±0.07
BMI	26.8±3.3	25.5±3.9				
**50–59 years**						
	(n = 246)	(n = 267)	(n = 227)	(n = 230)	(n = 441)	(n = 444)
Workload (Watts)	98.8±19.9	66.8±17.5	116.5±21.2	79.1±18.2	171.4±28.5	116.7±23.0
f_c_ (beats·min^−1^)	139±16	145±16	151±16	156±15	176±13	176±11
%f_cpeak_	78.3±7.7	82.2±7.1	85.2±7.3	88.4±6.4		
%VO_2peak_	67.9±10.6	72.3±10.2	75.6±10.4	80.6±9.6		
f_B_ (1·min^−1^)	26±5	28±5	29±6	30±6	42±8	40±7
V_E_ (L·min^−1^)	62.0±14.4	44.3±9.9	75.0±18.5	52.6±13.2	118.7±21.6	77.2±14.0
V_T_ (V_E_·f_B_^−1^)	2.40±0.45	1.61±0.30	2.58±0.45	1.74±0.31	2.83±0.45	1.92±0.30
VCO_2_ (L·min^−1^)	2.17±0.46	1.50±0.32	2.57±0.52	1.75±0.34	4.05±0.71	2.60±0.46
VO_2_ (L·min^−1^)	2.46±0.48	1.72±0.31	2.78±0.52	1.90±0.33	3.61±0.60	2.35±0.38
R (CO_2_·VO_2_^−1^)	0.90±0.06	0.88±0.08	0.95±0.07	0.94±0.08	1.12±0.07	1.11±0.07
BMI	27.3±3.1	25.9±3.6				
**60–69 years**						
	(n = 203)	(n = 167)	(n = 186)	(n = 144)	(n = 313)	(n = 284)
Workload (Watts)	87.4±20.5	57.3±17.4	103.9±21.1	70.1±18.4	157.7±32.7	108.6±23.4
f_c_ (beats·min^−1^)	131±16	140±19	143±17	150±18	169±14	168±13
%f_cpeak_	77.9±8.5	83.8±8.4	84.6±8.0	89.3±7.5		
%VO_2peak_	68.4±11.1	74.2±9.7	76.5±11.0	81.5±9.1		
f_B_ (1·min^−1^)	26±5	28±5	29±6	30±5	41±7	38±6
V_E_ (L·min^−1^)	58.5±13.8	42.9±10.0	68.8±16.3	49.4±11.1	109.0±20.7	70.0±12.5
V_T_ (V_E_·f_B_^−1^)	2.27±0.46	1.57±0.31	2.42±0.43	1.68±0.33	2.70±0.47	1.85±0.30
VCO_2_ (L·min^−1^)	1.95±0.43	1.43±0.31	2.26±0.49	1.65±0.35	3.57±0.68	2.32±0.42
VO_2_ (L·min^−1^)	2.24±0.45	1.62±0.32	2.49±0.48	1.77±0.32	3.23±0.57	2.14±0.36
R (CO_2_·VO_2_^−1^)	0.88±0.05	0.88±0.06	0.93±0.09	0.93±0.10	1.10±0.07	1.08±0.08
BMI	26.9±2.9	26.2±3.7				
**+70 years**						
	(n = 66)	(n = 60)	(n = 60)	(n = 52)	(n = 103)	(n = 99)
Workload (Watts)	67.3±25.2	42.5±18.4	83.4±26.9	57.3±18.6	133.8±32.2	94.6±25.6
f_c_ (beats·min^−1^)	127±14	133±18	136±18	145±20	158±17	158±19
%f_cpeak_	80.9±7.7	84.2±7.2	86.2±8.0	90.0±7.7		
%VO_2peak_	70.4±10.9	74.4±9.9	77.6±11.0	81.4±9.1		
f_B_ (1·min^−1^)	26±5	28±5	28±6	30±5	37±7	36±5
V_E_ (L·min^−1^)	52.2±14.4	37.8±8.4	60.1±17.1	43.7±10.6	90.7±21.0	58.7±16.5
V_T_ (V_E_·f_B_^−1^)	2.01±0.39	1.37±0.25	2.14±0.42	1.47±0.28	2.46±0.38	1.62±0.41
VCO_2_ (L·min^−1^)	1.64±0.43	1.19±0.25	1.92±0.50	1.38±0.32	2.90±0.67	1.89±0.42
VO_2_ (L·min^−1^)	1.91±0.44	1.39±0.29	2.14±0.50	1.52±0.32	2.71±0.56	1.80±0.36
R (CO_2_·VO_2_^−1^)	0.88±0.06	0.88±0.05	0.92±0.06	0.93±0.08	1.06±0.08	1.04±0.07
BMI	26.0±2.7	26.2±3.9				

Data are presented as arithmetic mean ± SD. Workload: treadmill exercise load, fc: cardiac frequency, fB: breathing frequency, VE: minute ventilation, VT: tidal volume, VCO2: expired carbon dioxcide, VO2: oxygen uptake, R: respiratory exchange ratio, BMI: body mass index, VThan: ventilatory anaerobic threshold, RCP: respiratory compensation point.

Women had approximately 32% (p<0.001) lower V_Tpeak_ than men. For both sexes the highest V_Tpeak_ was found among those aged 30–49 years, despite no differences in stature compared to the youngest age groups. In both sexes we observed an average 4% (p<0.05) and 11% (p<0.001) drop in V_Tpeak_ per decade in age groups 40–69 years, and between the 2 most senior age groups, respectively (Table 2). The highest breathing frequency (f_B_) in both men and women, 50±9 breaths·min^−1^ and 47±7 breaths·min^−1^, respectively, was found in the youngest age group (20–29 years), with an average 5% (p<0.05) decrease per subsequent decade (Table 2).

Stratified by height an 11% (p<0.01) rise in V_Epeak_ and V_Tpeak_ was observed per 10 cm increased height, in both sexes (Table 3).

**Table 3:** Peak respiratory variables in the HUNT 3 fitness study stratified by sex and height.

	Men	Women
**150–159 cm**		
		(n = 269)
V_E_ (L·min^−1^)		71.0±15.8
V_T_ (V_E_·f_B_^−1^)		1.68±0.29
f_B_ (1·min^−1^)		42±8
**160–169 cm**		
	(n = 128)	(n = 1146)
V_E_ (L·min^−1^)	104.7±22.4	81.0±16.6
V_T_ (V_E_·f_B_^−1^)	2.40±0.37	1.93±0.30
f_B_ (1·min^−1^)	44±9	42±7
**170–179 cm**		
	(n = 846)	(n = 432)
V_E_ (L·min^−1^)	117.3±23.8	90.1±16.7
V_T_ (V_E_·f_B_^−1^)	2.71±0.42	2.13 ±0.31
f_B_ (1·min^−1^)	44±8	42±8
**180–189 cm**		
	(n = 740)	(n = 13)
V_E_ (L·min^−1^)	132.0±23.6	92.5±21.6
V_T_ (V_E_·f_B_^−1^)	3.00±0.46	2.12±0.61
f_B_ (1·min^−1^)	44±8	44±16
**190–200 cm**		
	(n = 93)	
V_E_ (L·min^−1^)	144.7±26.6	
V_T_ (V_E_·f_B_^−1^)	3.32±0.50	
f_B_ (1·min^−1^)	43±9	

Data are presented as arithmetic mean ± SD. VE: ventilation, VT: tidal volume, fB: breathing frequency.

### Carbon dioxide (VCO_2_) elimination

Women displayed roughly 34% (p<0.001) lower VCO_2peak_ than men. Stratified by age the highest VCO_2peak_ was found in the youngest age groups. No significant differences in VCO_2peak_ were observed neither for men nor women between age groups 20–29 and 30–39 years, whereupon an approximate 6% (p<0.001) and 5% (p<0.001) decrease was observed between age groups 30–39 vs. 40–49 years, respectively. In subsequent age groups exponential reductions were observed, ending with an average 18% (p<0.001) lower peak VCO_2_ in the most senior age group compared with men and women aged 60–69 years (Table 2).

### Ventilatory anaerobic threshold (V_Than_)

The highest V_Than_ was observed in the youngest age groups. No statistical differences in V_Than_ was observed between the 3 youngest age groups (20–49 years) among both sexes, whereupon we observed an approximate 10% (p<0.001) lower V_Than_ per decade. V_Than_ was obtained at 75.2±10.7 and 76.7±9.4% of VO_2peak_ for men and women (20–29 years), respectively, which corresponds to approximately 88±7% of peak heart rate (f_cpeak_), with no major differences between age groups (Table 4).

**Table 4:** Cardiorespiratory variables in the HUNT 3 Fitness study stratified by sex, age groups.

	N	VO_2peak_ (L·min^−1^)	VO_2peak_ (mL·kg·min^−1^)	V_Than_ (L·min^−1^)	V_Than_(%VO_2peak_)	RCP (L·min^−1^)	RCP (%VO_2peak_)
**All**							
Men	1050	3.74±0.76	44.2±9.3	2.83±0.67	76.4±10.4	3.24±0.71	86.4±10.9
Women	1013	2.47±0.51	35.8±7.7	1.90±0.43	77.6±9.7	2.21±0.50	89.2±10.4
**20–29 years**							
Men	101	4.30 ±.73	54.0±8.8	3.20±0.75	75.2±10.7	3.59±0.69	85.6±10.3
Women	92	2.77±0.47	42.8±7.6	2.08±0.44	76.7±9.4	2.48±0.43	90.9±8.3
**30–39 years**							
Men	172	4.20±0.65	48.6±7.9	3.17±0.62	75.6±10.3	3.57±0.65	85.4±10.5
Women	203	2.74±0.50	39.6±7.0	2.07±0.45	75.6±9.8	2.44±0.52	88.9±12.3
**40–49 years**							
Men	264	4.00±0.62	46.6±8.0	3.04±0.63	76.6±11.0	3.44±0.67	85.5±11.0
Women	249	2.62±0.44	37.8±7.0	2.02±0.40	77.6±9.7	2.36±0.45	88.5±9.9
**50–59 years**							
Men	267	3.61±0.60	41.9±7.6	2.71±0.53	76.2±10.1	3.19±0.62	86.9±11.4
Women	259	2.35±0.38	33.7±5.7	1.82±0.36	78.2±9.4	2.12±0.42	89.1±11.2
**60–69 years**							
Men	182	3.23±0.57	38.4±6.9	2.48±0.49	77.5±10.1	2.85±0.54	88.4±10.8
Women	152	2.14±0.36	30.5±5.1	1.68±0.34	78.9±9.3	1.94±0.34	90.8±8.2
**+70 years**							
Men	64	2.71±0.56	34.2±7.1	2.05±0.48	77.4±9.8	2.37±0.49	87.4±9.6
Women	58	1.80±0.36	26.8±5.1	1.44±0.33	79.8±10.7	1.59±0.33	87.3±10.1

Data are presented as arithmetic mean ± SD. VO2peak: peak oxygen uptake, VThan: Ventilatory anaerobic threshold, RCP: respiratory compensation point.

### Respiratory compensation point (RCP)

The highest RCP was observed in the youngest age groups with roughly the same decline rate per decade as observed for V_Than_ (Table 4).

### Ventilatory efficiency at VO_2peak_ and at V_Than_

EqVO_2peak_ were similar between sexes and age groups. EqVCO_2peak_ was on average 1.2% (p<0.05) higher in women than in men. In men aged 20–59 years no differences were observed between subsequent age groups, upon which we observed a 4.4% (p<0.001) higher EqVCO_2peak_ for men aged 60–69 years compared to those aged 50–59 years. No differences were shown between the two oldest male age groups. In women we observed no differences in EqVCO_2peak_ between subsequent age groups throughout all decades. Comparing the youngest and oldest age groups an 8% (p<0.001) higher EqVCO_2peak_ was observed in the oldest age group, in both men and women (Table 5).

**Table 5:** Ventilatory equivalents at peak exercise and at ventilatory anaerobic threshold in the HUNT 3 fitness study.

	Men	Women
**20–29 years**		
	(n = 207)	(n = 246)
EqVO_2peak_ (V_Epeak_·VO_2peak_^−1^)	33.2±4.1	33.5±5.0
EqVCO_2peak_ (V_Epeak_·VCO_2peak_^−1^)	29.0±3.3	29.3±4.0
	(n = 69)	(n = 75)
EqVO_2VThan_ (V_EVThan_·VO_2VThan_^−1^)	24.9±3.6	26.3±3.5
EqVCO_2VThan_ (V_EVThan_·VCO_2VThan_^−1^)	26.2±2.8	27.5±2.7
**30–39 years**		
	(n = 342)	(n = 417)
EqVO_2peak_ (V_Epeak_·VO_2peak_^−1^)	32.9±4.3	33.8±4.6
EqVCO_2peak_ (V_Epeak_·VCO_2peak_^−1^)	28.6±3.4	29.6±3.7
	(n = 134)	(n = 181)
EqVO_2VThan_ (V_EVThan_·VO_2VThan_^−1^)	25.0±2.9	26.9±4.2
EqVCO_2VThan_ (V_EVThan_·VCO_2VThan_^−1^)	26.7±2.4	28.5±3.6
**40–49 years**		
	(n = 593)	(n = 604)
EqVO_2peak_ (V_Epeak_·VO_2peak_^−1^)	33.2±4.4	33.8±4.6
EqVCO_2peak_ (V_Epeak_·VCO_2peak_^−1^)	29.3±3.6	29.9±3.8
	(n = 265)	(n = 259)
EqVO_2VThan_ (V_EVThan_·VO_2VThan_^−1^)	26.3±3.8	27.5±4.6
EqVCO_2VThan_ (V_EVThan_·VCO_2VThan_^−1^)	28.0±3.3	29.3±3.9
**50–59 years**		
	(n = 579)	(n = 592)
EqVO_2peak_ (V_Epeak_·VO_2peak_^−1^)	33.1±4.8	33.1±4.8
EqVCO_2peak_ (V_Epeak_·VCO_2peak_^−1^)	29.5±4.0	30.0±4.1
	(n = 241)	(n = 245)
EqVO_2VThan_ (V_EVThan_·VO_2VThan_^−1^)	26.6±3.9	27.0±3.5
EqVCO_2VThan_ (V_EVThan_·VCO_2VThan_^−1^)	28.3±3.6	29.0±3.1
**60–69 years**		
	(n = 401)	(n = 373)
EqVO_2peak_ (V_Epeak_·VO_2peak_^−1^)	33.9±5.3	33.0±5.0
EqVCO_2peak_ (V_Epeak_·VCO_2peak_^−1^)	30.8±4.5	30.6±4.5
	(n = 203)	(n = 158)
EqVO_2VThan_ (V_EVThan_·VO_2VThan_^−1^)	27.5±4.1	27.4±3.6
EqVCO_2VThan_ (V_EVThan_·VCO_2VThan_^−1^)	29.7±4.1	29.4±3.3
**+70 years**		
	(n = 137)	(n = 134)
EqVO_2peak_ (V_Epeak_·VO_2peak_^−1^)	33.7±5.4	32.8±8.4
EqVCO_2peak_ (V_Epeak_·VCO_2peak_^−1^)	31.6±4.5	31.4±7.8
	(n = 69)	(n = 56)
EqVO_2VThan_ (V_EVThan_·VO_2VThan_^−1^)	28.6±3.9	28.5±3.9
EqVCO_2VThan_ (V_EVThan_·VCO_2VThan_^−1^)	31.3±4.0	31.0±3.0

Data are presented as arithmetic mean ± SD. EqVO2 and EqVCO2: ventilatory efficiency.

EqVO_2Than_ was on average 3% (p<0.001) higher in women than in men. EqVO_2Than_ was similar and lowest in the two youngest male age groups (20–29 and 30–39 years). We observed a 5.2% (p<0.05) higher EqVO_2Than_ for men aged 40–49 years compared to those aged 30–39 years, with no differences between the subsequently older male age groups (40–49 through +70 years). In women no differences were observed between subsequent age groups throughout all decades. When comparing the youngest and oldest age groups a 12% (p<0.05) higher EqVO_2Than_ was observed in the oldest age group, among both men and women (Table 5).

EqVCO_2Than_ was on average 2.5% (p<0.001) higher in women compared to men. EqVCO_2Than_ was alike and lowest in the two youngest male age groups (20–29 and 30–39 years), whereas a 4.9% (p<0.01) higher EqVCO_2Than_ was observed in men 40–49 years compared to the 30–39 years group. No differences were found between men aged 40–49 years and 50–59 years, whereupon an average 5.3% (p<0.05) higher EqVCO_2Than_ was observed per decade between the three oldest male age groups. In women aged 20–69 years no differences in EqVCO_2Than_ was observed between subsequent age groups, upon which a 5.4% (p<0.05) higher EqVCO_2Than_ was observed in the oldest age group compared to those 60–69 years. Comparing the youngest and the oldest age groups showed a 16% (p<0.001) higher EqVCO_2Than_ in the oldest group, in both men and women (Table 5).

### EqVCO_2VThan_, among the 3 oldest age groups, stratified by fitness quartiles

In those aged 50–59 years there was a 7.2% (p<0.05) and 8.2% (p<0.001) increase between the most fit (Q_1_) and least fit (Q_4_), men and women, respectively. The middle (60–69 years) and the most senior groups (+70 years) had increases of 13.4% (p<0.001; men) vs. 13.1% (p<0.001; women), and 16.4% (p<0.01; men) with no significant differences among women, between the fittest (Q_1_) and least fit (Q_4_), respectively (Table 6).

**Table 6:** Ventilatory efficiency and oxygen uptake presented in fitness quartiles: The HUNT 3 fitness study.

	Men		Women	
	V_EVThan_•VCO_2VThan_^−1^	VO_2peak_ (mL•kg•min^−1^)	V_EVThan_•VCO_2VThan_^−1^	VO_2peak_ (mL•kg•min^−1^)
**50–59 years**				
	(n = 241)		(n = 242)	
Q_1_	27.6±3.8	51.8±4.3	28.2±2.8	41.3±2.9
Q_2_	27.5±2.9	44.0±2.9	28.1±2.8	35.1±1.3
Q_3_	28.5±3.0	39.2±1.5	29.1±2.5	31.5±1.1
Q_4_	29.6±4.4	32.6±3.0	30.5±3.5	26.8±2.4
**60–69 years**				
	(n = 202)		(n = 158)	
Q_1_	28.4±2.6	47.6±4.1	28.2±2.9	37.1±3.0
Q_2_	29.5±3.7	40.2±1.4	29.5±3.0	32.1±0.9
Q_3_	29.5±3.8	35.5±1.2	28.7±2.7	28.7±1.0
Q_4_	32.2±5.5	30.3±3.0	31.9±3.9	24.2±2.4
**+70 years**				
	(n = 69)		(n = 56)	
Q_1_	29.2±3.8	43.5±4.4	30.1±3.0	33.4±3.7
Q_2_	31.0±3.1	35.9±1.4	30.8±2.6	27.9±0.9
Q_3_	30.7±3.6	31.4±1.4	30.9±2.3	24.8±0,8
Q_4_	34.0±4.2	25.6±2.5	32.7±3.4	20.8±2.0

Data are presented as arithmetic mean ± SD. VEVThan•VCO2VThan-1: ventilatory efficiency at ventilatory anaerobic threshold. Q1-4: quartiles of ventilatory efficiency and oxygen uptake.

### Estimating key cardio pulmonary parameters from non-exercise prediction models

Prediction equations for V_Epeak_, VCO_2peak_ and V_Than_ were derived from non-exercise variables, including weight, height, age and sex. Weight and age proved negligible in predicting V_Tpeak_, as did weight and height in predicting EqVCO_2VThan_ and EqVO_2VThan_, hence these variables were excluded from the respective models. For all models gender should be substituted with 1 or 2 for men and women, respectively. The final regression models are presented in table 7. Non-exercise prediction models for VO_2peak_ are previously published from the HUNT 3 fitness material [33].

**Table 7:** Multiple linear regression models for predicting key cardio respiratory variables from non-exercise variables: The HUNT 3 fitness study.

Equations	R^2^	SEE
V_Epeak_ (L•min^−1^) = 16.843+ (0.178 x weight) + (0.876 x height) − (0.762 x age) − (28.171 x gender)	0.67	17.5
VCO_2peak_ (L•min^−1^) = 1.264+ (0.012 x weight + (0.025 x height − (0.031 x age) − (0.981 x gender)	0.70	0.59
V_Than_ (L•min^−1^ of VO_2_) = (0.01 x weight) + (0.016 x height) − (0.017 x age) − (0.581 x gender) +0.514	0.59	0.47
V_Tpeak_ (L) = (0.032 x height) − (0.468 x gender) −2.379	0.64	0.37
EqVCO_2VThan_ = 23.897+ (0.072 x age) + (0.826 x gender)	0.08	3.47
EqVO_2VThan_ = 23.148+ (0.050 x age) + (0.808 x gender)	0.04	3.91

VEpeak: peak ventilation, VECO2: peak expiration of carbon dioxide, VThan: ventilatory anaerobic threshold, VTpeak: peak tidal volume, EqVCO2VThan and EqVO2VThan: ventilatory efficiency at ventilatory anaerobic threshold, R2: coefficient of variation, SEE: standard error of the estimate.

### Association between EqVCO_2VThan_ and age

**Figure 1:**
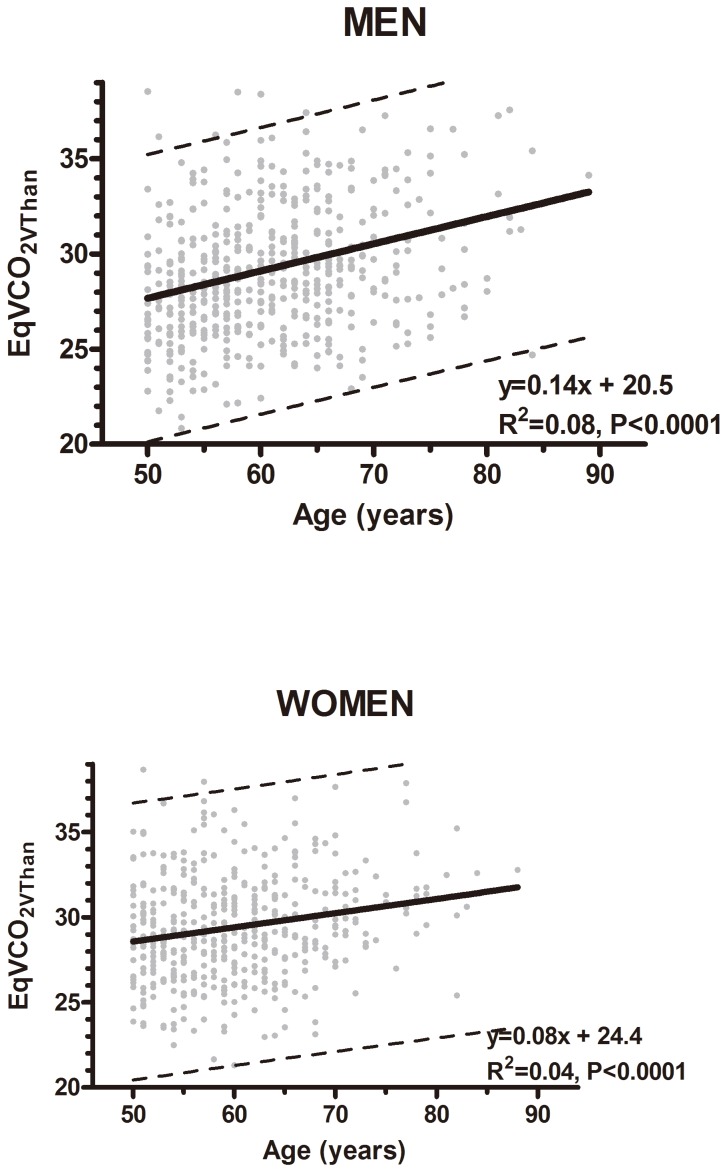
Correlations between EqVCO2VThan (ventilatory efficiency at ventilator anaerobic threshold) and age groups: The HUNT 3 fitness study.

Figure 1 displays the relationship between EqVCO_2VThan_ and age, with relative low, but statistical strong correlations r = 0.27 (p<0.0001) and r = 0.18 (p<0.001) among men and women, respectively.

### Associations between V_E_ and VCO_2_

**Figure 2:**
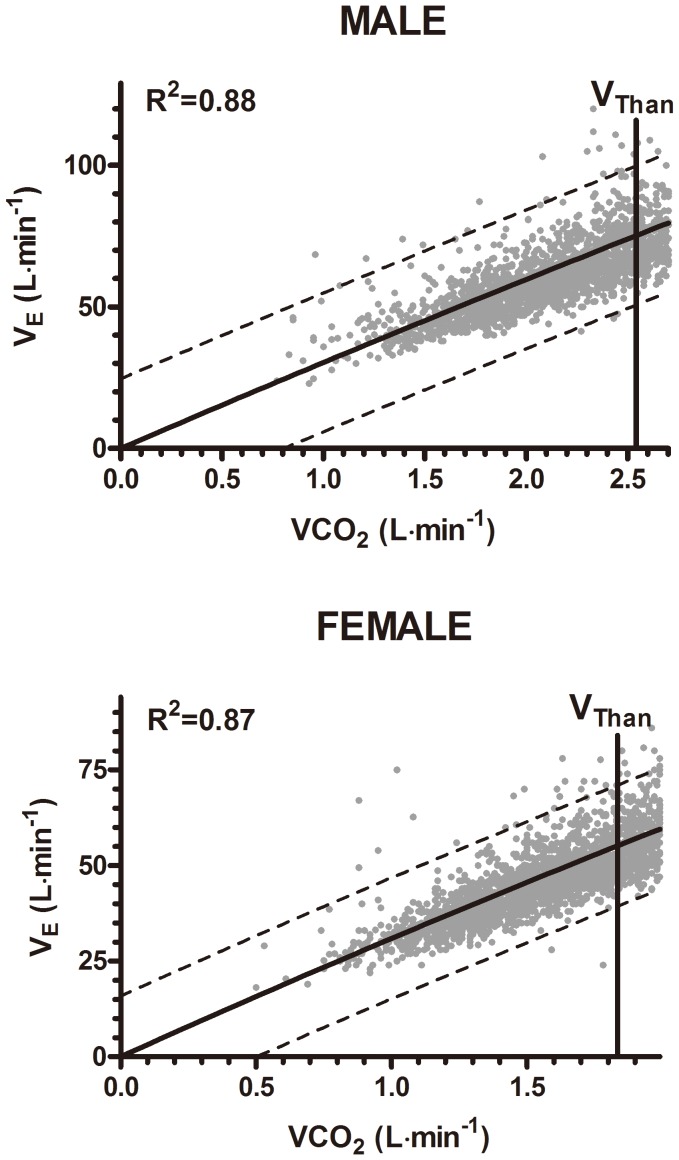
Correlations between V_E_ and VCO_2_ from test start up to V_Than_: The HUNT 3 fitness study.

Figure 2 show the relationship between V_E_ and VCO_2_ from start of test until V_Than_, with strong correlations (men: r = 0.94; women: r = 0.93).

## Discussion

This is the largest European cardio-respiratory reference material in healthy men and women aged 20–90 years. Previous papers mostly present smaller, selected age groups [15], [18], [34]–[40], or only male populations [23], [25], [41]. Hence, this study will serve as a useful addition to previous research.

### Sex and age group differences in peak ventilation (V_Epeak_) and tidal volume (V_Tpeak_)

In this study women had approximately 34% and 32% lower V_Epeak_ and V_Tpeak_, respectively, and a 4% lower peak f_Bpeak_ than men. This is in agreement with other population-based studies on V_Epeak_ [39], [42]–[44], V_Tpeak_ and f_Bpeak_ [22], [45], and as expected as women have smaller lung size and dynamic lung function volumes than men, also after adjusting for differences in stature [46]. We observed 6–30% higher V_Epeak_ among men and women compared to that seen in Brazilian [42] (n = 3992), American [43] (n = 988) and French [44] (n = 150) populations, as well as in small sample size studies [22], [45], [47], [48]. Yet, a Norwegian study [49] (n = 759) displays V_Epeaks_ fairly consistent with ours. Hence, there might be population differences, which highlight the need of reference data in different populations.

Lower V_Epeak_ with increasing age is consistent with findings in Brazilian [42] (n = 3992), Israeli [23] (male  = 1424), Canadian [39] (n = 100), [25] (male  = 816) and French [44] (n = 150) studies, and in line with an age attenuation in dynamic lung function largely attributed to decreased elastic recoil [50], [51].

In this study the highest V_Tpeak_ was observed among the 30–49 year groups, in both men and women, with a decrease in subsequent age groups. These findings are unexpected, since the highest V_T_ should be in the youngest age group, with deterioration between subsequently older age groups [50], [51]. Our findings could be explained by the relative low sample size in the youngest age group. Contrary to us a Canadian [39] (n = 100) and Israeli [23] (n = 1424) study presented their highest V_Tpeak_ in the youngest age groups (15–25 yrs). Interestingly, while the Canadian study displayed the same male average V_Tpeak_ as us, they have significantly lower V_Epeak_, signaling a necessarily lower f_Bpeak_ (not displayed).

### Association between V_T_ and V_E_ below and above V_Than_

On the initial sub maximal workloads the V_T_ vs. V_E_ slope displays a steeper gradient than towards test termination. This is to be expected, since it is well established that V_T_ increases steeper than f_B_ below V_Than_, whereas f_B_ mostly accounts for the increase in V_E_ at workloads above V_Than_ [47], [52].

### Sex and age group differences in V_Than_ and RCP

We observed V_Than_ at an average 77% of VO_2peak_, in both sexes, and in line with other studies (n = 204–3992) [24], [40], [42], [53], [54] minor sex differences. Previous studies report V_Than_ at significantly lower fractions (49–70% of VO_2peak_) [23], [24], [40], [42], [53], [54], or more consistent to that observed by us [25], [55], [56]. Differences are most likely caused by use of different methods and analyzing approaches applied in the different studies, which makes direct comparisons difficult [23], [25], [42], [55], [56].

In this study V_Than_ was observed at ≈75% in the youngest age groups and at ≈80% of VO_2peak_ in the oldest age groups, in both men and women, with significant differences (p<0.05) between the youngest and the 60–69 year age group. Age related increase in V_Than_ (as percent of VO_2peak_) is reported in previous studies as well [23]–[25], [42], [44], [53], [54], [57]. This is to be expected since V_Than_ (L·min^−1^) declines at a slower rate than VO_2peak_ (L·min^−1^) with increasing age [58], [59], and consequently occurs at a higher percent of VO_2max/peak_ [60]. This is suggested to be, at least partly, due to changes in skeletal muscle composition associated with increasing age, with the selective loss of type ΙΙ fibers and therefore a relative increase in type Ι fibers [61]. Contrary to this Lenti and colleagues [34] report a decrease in percent V_Than_ in a trained senior group (n = 16), compared to their young trained, whereas the untrained groups are consistent with our findings (n = 16). The small sample size taken into account, their data must be interpreted with caution.

RCP was observed at 86% and 90% of VO_2peak_, among men and women, respectively. This is consistent with the findings of several other studies [18], [35], [37]. However, direct comparisons are difficult due to their small sample sizes (n = 9–22) and the use of different measuring methods.

### Ventilatory efficiency stratified by sex

In line with previous studies [17], [22] we observed similar EqVO_2peak_ in men and women. Wasserman [52] suggests that EqVCO_2_ should be determined at V_Than_, or between V_Than_ and RCP as V_E_ is least variable in this range. Our submaximal level 2 measurements are close to V_Than_ and we observed slightly higher (p<0.001) EqVCO_2VThan_ in women than men, hence indicating less efficient ventilation in women. These observations are in agreement with previous studies [19], [20], [62]. Women's lower ventilatory efficiency might be explained by differences in ventilatory stimuli (e.g. [H^+^], [K^+^]), metaboreceptors, and central command [63], [64].

### Ventilatory efficiency at V_Than_ stratified by age groups

We observed deterioration in ventilatory efficiency, both in EqVO_2Than_ (p<0.05) and EqVCO_2Than_ (p<0.001), between the youngest and oldest age groups. This is in agreement with the findings of other studies (n = 69–474) [19], [20], [57], [62]. It is presently uncertain which factors are responsible for diminished ventilatory efficiency during exercise with increasing age [65]. Clearly, increased dead space might be a major contributing factor, as well as the lung's mechanical limitation to airflow, which deteriorate as the lung loses elastic recoil with increasing age [66]. In women evidence points to decreased leg muscle strength as a contributing factor [65]. Other suggestions are factors linked to muscle afferent excitability as a result of fiber type shifts [67], and neuromuscular alterations with growing age [68].

### EqVCO_2VThan_ stratified by fitness quartiles

Individuals in the three oldest age groups (50–59, 60–69, +70 years) in the present study are more likely to be referred to clinical exercise testing than younger age groups, and have been studied in more depth than other age groups when it comes to EqVCO_2VThan_. Interestingly, among individuals in these age groups approximately 25% had EqVCO_2VThan_ higher than 30. EqVCO_2VThan_ <30 is considered normal with a possible increase among older age groups [1]. In these age groups we observed minor differences in EqVCO_2VThan_ between those that were among the three first quartiles of fitness (VO_2peak_ quartiles), whereupon we observed a significant drop in ventilatory efficiency (hence, an increase in EqVCO_2VThan_) in those categorized as being least fit (Q_4_) in both sexes and all three age groups. All fitness quartiles in the oldest age group (+70 years), and the least fit quartiles in the two younger age groups (50–59, 60–69 years) had EqVCO_2VThan_ ≈>30, which could be caused by high dead space ventilation due to diminished alveoli perfusion [19]. More importantly, diminished ventilatory efficiency can reflect disease severity and prognosis in several patient groups including chronic obstructive pulmonary disease, pulmonary arterial hypertension, hypertrophic cardiomyopathy and interstitial lung disease [1], [15], and are displayed in the range 41–60 in more severe cases of congestive heart failure [15]. Although we cannot totally exclude the possibility of unknown diseases in some of our participants, the self-report and medical interview, adhering to our inclusion criteria, provides a healthy population. Therefore seen in context of our sample size our findings may represent normal ventilatory efficiency values for the oldest age groups and least fit population.

### Non-exercise prediction models for key cardio-respiratory variables

There is a plethora of VO_2peak_ prediction models. However, models on other key cardio-respiratory variables are less abundant. The accuracy of previously published models on VO_2peak_ from the HUNT 3 fitness data (Men: 12.8%, Women: 14.3%) [33] is in fair agreement with previous large sample studies (Jurca 2005). Smaller sample studies [69]–[71] with uniform populations [72], [73] show accuracies in the range ±7–17%. Also V_Than_ accuracy (±19.9%) is approximately the same compared to 7 previous small-scale studies [40]. Our precision of V_Epeak_ prediction (±17.2%) is in agreement with a large study of males [23], contrasted by a small study [39] showing 28% accuracy. However, it was hard to compare our V_Tpeak_ accuracy (±15.5%) with others [23], since key data was not presented. The 12.1% accuracy in predicting EqVCO_2VThan_ was similar to a small sample study [62]. Our prediction models will provide a rough estimation of these key variables, regardless of gender and age. Moreover, the models use non-exercise variables that are easy to measure, thus making these models easy to use in both clinical settings and for recreational athletes.

### Association between ventilatory efficiency and workload

The slope of the ventilatory equivalent for oxygen (EqVO_2_·W^−1^) increases with rising workload (w), which demonstrates reduced ventilatory efficiency as the workload increases. This is supported by two former case studies [30], [52]. More interestingly the EqVCO_2_·W^−1^ displays a gradient close to zero, which indicates a constant ventilatory efficiency throughout the incremental workload, and is in fair agreement with Wasserman and colleagues [52], yet, contradicted by another study [30] that presents an increase in EqVCO_2_ as the workload approaches peak. However, it is noteworthy that these studies are based on single case observations, and thus difficult to compare with our findings.

### Strengths and limitations

The large sample size, inclusion of men and women, wide age distribution and cardio-respiratory measurements up to the true VO_2max_ makes this study robust. The lack of spirometry data limits the assessment of ventilatory parameters. Also this study may be subject to bias due to self-selection caused by the low participation rate. However, almost all those invited to the current Fitness study from the large HUNT study agreed to participate in the fitness test. Due to limited capacity at the test sites resulting in long waiting lines, some potential participants chose to withdraw their participation from the study. Those who finally participated in the study could thus be healthier than those who quit or declined participation. However, a comparison of the participants in the fitness study with a healthy sample of the total HUNT population (i.e. free from cardiovascular or pulmonary diseases, cancer, or sarcoidosis) confirmed that the fitness participants did not considerably differ from other healthy HUNT participants [26].

## Conclusions

The selection of key cardio-respiratory variables combined with an age and sex stratified large sample size makes this material unique. The inconsistencies between this and earlier research, and the scarce availability of large sample size materials support the usefulness of a large reference material as presented in this study. The HUNT 3 Fitness study presents the largest European reference material of cardio-respiratory variables in healthy men and women aged 20–90 years. Our material establishes normal values for, and associations between, as well as providing prediction models for these key cardio-respiratory parameters.
